# Tumor necrosis factor-α and interferon-γ stimulate MUC16 (CA125) expression in breast, endometrial and ovarian cancers through NFκB

**DOI:** 10.18632/oncotarget.7652

**Published:** 2016-02-24

**Authors:** Micaela Morgado, Margie N. Sutton, Mary Simmons, Curtis R. Warren, Zhen Lu, Pamela E. Constantinou, Jinsong Liu, Lewis LW. Francis, R. Steven Conlan, Robert C. Bast, Daniel D. Carson

**Affiliations:** ^1^ Department of BioSciences, Wiess School of Natural Sciences, Rice University, Houston, TX, USA, 77251; ^2^ Department of Experimental Therapeutics, Division of Cancer Medicine, The University of Texas M.D. Anderson Cancer Center, Houston, TX, USA, 77030; ^3^ The University of Texas Graduate School of Biomedical Sciences, The University of Texas Health Science Center at Houston, Houston, TX, USA, 77030; ^4^ Department of Stem Cell and Regenerative Biology, Harvard University, Cambridge, MA, USA, 02138; ^5^ Department of Pathology, The University of Texas M.D. Anderson Cancer Center, Houston, TX, USA, 77030; ^6^ Department of Genetics, The University of Texas MD Anderson Cancer Center Houston, TX, USA, 77030; ^7^ Swansea University Medical School, Singleton Park, SA2 8PP, Swansea, Wales, UK

**Keywords:** MUC16, CA125, cytokine, NFκB, cancer

## Abstract

Transmembrane mucins (TMs) are restricted to the apical surface of normal epithelia. In cancer, TMs not only are over-expressed, but also lose polarized distribution. MUC16/CA125 is a high molecular weight TM carrying the CA125 epitope, a well-known molecular marker for human cancers. MUC16 mRNA and protein expression was mildly stimulated by low concentrations of TNFα (2.5 ng/ml) or IFNγ (20 IU/ml) when used alone; however, combined treatment with both cytokines resulted in a moderate (3-fold or less) to large (> 10-fold) stimulation of MUC16 mRNA and protein expression in a variety of cancer cell types indicating that this may be a general response. Human cancer tissue microarray analysis indicated that MUC16 expression directly correlates with TNFα and IFNγ staining intensities in certain cancers. We show that NFκB is an important mediator of cytokine stimulation of MUC16 since siRNA-mediated knockdown of NFκB/p65 greatly reduced cytokine responsiveness. Finally, we demonstrate that the 250 bp proximal promoter region of MUC16 contains an NFκB binding site that accounts for a large portion of the TNFα response. Developing methods to manipulate MUC16 expression could provide new approaches to treating cancers whose growth or metastasis is characterized by elevated levels of TMs, including MUC16.

## INTRODUCTION

Mucins are high molecular weight glycoproteins that are normally found on apical surfaces of epithelial organs such as trachea, stomach, and reproductive organs [1], where they serve various functions including protection against pathogenic infections, hydration and cellular signaling [2]. To date, 20 mucin genes have been identified and are classified based on the presence of large, heavily O-glycosylated, tandem repeat motifs [3]. Mucins can be membrane bound or secreted depending on the presence of a membrane spanning region [2]. Of the cell surface bound mucins, MUC1, MUC4 and MUC16 are the best characterized [4].

Mucins are a class of molecules that aid in mucosal defense by providing a large physical cell surface barrier to pathogens and tissue-degrading enzymes [1]. Mucins normally function to protect and lubricate the epithelium, but are overexpressed in various cancers [5], are frequently used as diagnostic markers [6, 7], and are being considered as therapeutic targets [8]. Alterations in transmembrane mucin expression or glycosylation promote the development of cancer and stimulate cell growth, differentiation and invasion [9, 10]. MUC16 (also known as the serum tumor marker, CA 125) is the largest transmembrane mucin being 2–5 MDa including O-linked and N-linked glycosylation. The polypeptide backbone of MUC16 contains 22,000 amino acids [11, 12], including an N-terminal tandem repeat region composed of 18–60 repeats of 156 amino acids each, and a C-terminal region with a short cytoplasmic tail [4, 12]. Intact MUC16 is the largest membrane glycoprotein known, towering over even other large cell surface mucins like MUC1 and MUC4. As such, it likely represents the initial point of contact with other cells and matrices. MUC16 is believed to play important roles not only in normal contexts such as reproduction, but also in pathological states including cancer and mucosal infections [13–15]. MUC16 has been used as a tumor marker for over thirty years due to its overexpression in ovarian and other cancers, yet little is known about its regulation. The importance of MUC16 in the diagnosis, progression and therapy of ovarian cancer, and its overexpression in other cancers, demands a need for research on the regulation of this mucin.

Eighty to 90 percent of all cancers originate in epithelial tissue. Membrane bound mucins are overexpressed in many of these cancers as well as in other pathological conditions [2]. The causes of mucin overexpression are not always clear, but include gene duplication [16], and mucin gene responsiveness to factors in the tumor microenvironment [17]. High levels of transmembrane mucins on tumor cells protect these cells from attack by the host immune system as well as from the actions of cytotoxic drugs [17].

The regulation of expression of certain mucins, particularly MUC1, has been well studied and is markedly stimulated by cytokines [18–21]. Inflammatory events trigger cytokine release by immune cells, which invade tumor-associated stroma. Inflammation and cytokine production are one of the proposed “hallmarks of cancer” implicating their significant role in tumorigenesis [22]. Furthermore, it has been shown that NFκB directly binds to the *MUC1* promoter to activate gene transcription (12). NFκB generally plays a key role as a mediator of inflammatory responses, and also has been found to play a crucial role in many steps of cancer initiation and progression [23]. In spite of the existing detailed information on the molecular regulation of MUC1, little is known about regulation of *MUC16* gene expression [24, 25].

At least 20% of all cancers are associated with chronic inflammation, typified by a cytokine-rich environment [26]. This inflammation is most often assessed by histological detection of tumor-associated or infiltrating, cytokine-producing immune cells. Even cancers that do not develop from chronic inflammation often contain high levels of cytokines [26]. Macrophages from tumors secrete inflammatory cytokines including TNFα and IFNγ. TNFα has a tumor-promoting role (19), and TNFα expression generally increases with tumor stage (20). Also, high plasma levels of TNFα correlate with higher tumor stage (21). On the other hand, IFNγ has dual roles with both pro-inflammatory and anti-inflammatory properties [27]. Both cytokines have significant physiological importance in regulating immune responses and inflammation. In this study, we link the expression of MUC16 to stimulation by TNFα and IFNγ through NFκB in cell culture and in pathological specimens.

## RESULTS

### Basal MUC16 mRNA levels in various cell types differ among normal epithelial cells derived from breast, ovarian and endometrial cancers

**Figure 1:**
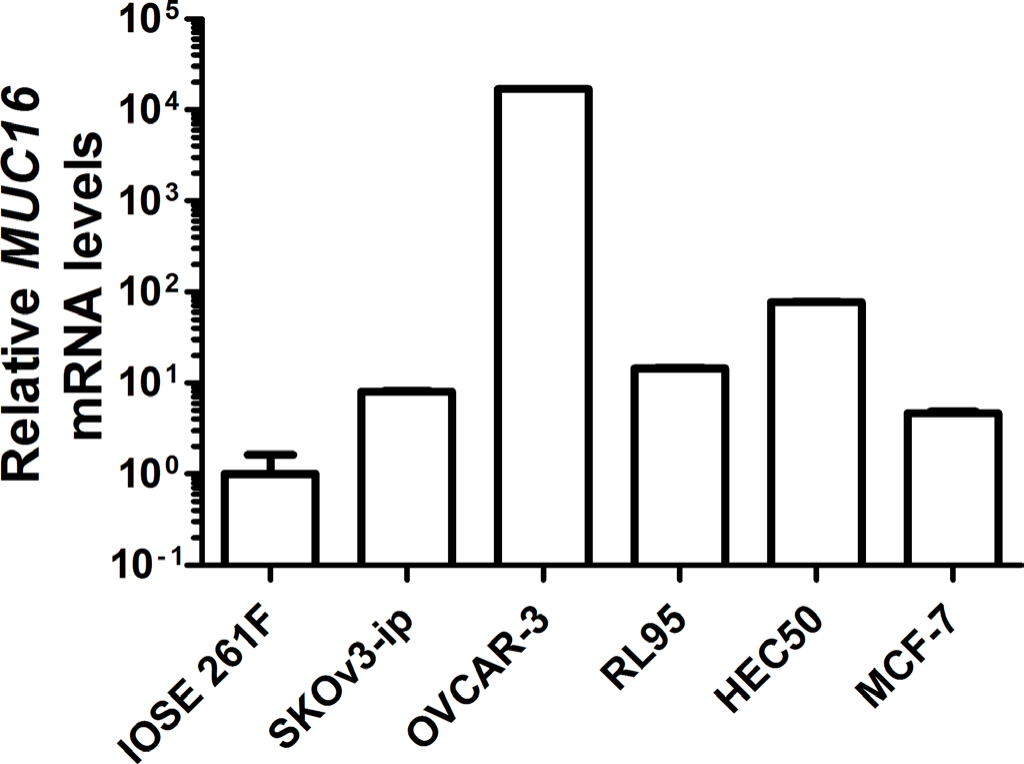
Basal *MUC16* mRNA levels in various epithelial cell types *MUC16* mRNA levels were measured *via* quantitative qRT-PCR relative to the mRNA levels for *ACTB* in the indicated cell lines as described in Materials and Methods. IOSE 261F is the cell line with the lowest basal MUC16 mRNA levels and its value was arbitrarily set to 1 for comparison. Although error bars are not evident in all cases, triplicate independent determinations were performed in each case with variation < 5% among samples. In order to express all values on the same graph due to the very high basal levels of MUC16 expressed by OVCAR-3 cells a log base 10 scale was used for the Y-axis.

Initially, we determined basal *MUC16* mRNA levels in a series of epithelial cells derived from female reproductive tissues: IOSE 261F (Table 1 and Figure 1) (a normal ovarian epithelial cell type), SKOv3-ip (Table 1 and Figure 1), and OVCAR-3 (Table 1 and Figure 1), moderately and poorly differentiated ovarian cancer cells, respectively, which displayed moderate (SKOv3-ip) and very high (OVCAR-3) basal levels of *MUC16* mRNA; RL95-2 and HEC50, moderately and poorly differentiated cells, respectively, derived from endometrial adenocarcinomas with moderate basal levels of *MUC16* (Table 1 and Figure 1); and MCF-7 (breast cancer), which displayed very low basal levels of *MUC16* (Table 1 and Figure 1).

**Table 1:** Cell types used in the current study

Cell line	Tissue source	Type	Level of differentiation	References
IOSE 261F	Ovary-surface epithelium	Normal	Well	Leung, E.H., et al. 2001 [48]
SKOv3-ip	Ovary: ascites	Adenocarcinoma	Moderate	Hua, W., et al. 1995 [49]
OVCAR-3	Ovary: ascites	Adenocarcinoma	Poor	Hamilton, T.C., et al. 1983 [50]
RL95-2	Uterus: endometrium	Adenocarcinoma	Moderate	Way, D.L., et al. 1983 [51]
HEC-50	Uterus: endometrium	Adenocarcinoma	Poor	Kassan, S., et al. 1989 [52]
MCF-7	Breast: mammary gland	Adenocarcinoma	Moderate	Soule, H.D., et al. 1973 [53]

### TNFα and IFNγ stimulate MUC16 mRNA levels in MCF-7 breast cancer cells in a dose-dependent manner

**Figure 2:**
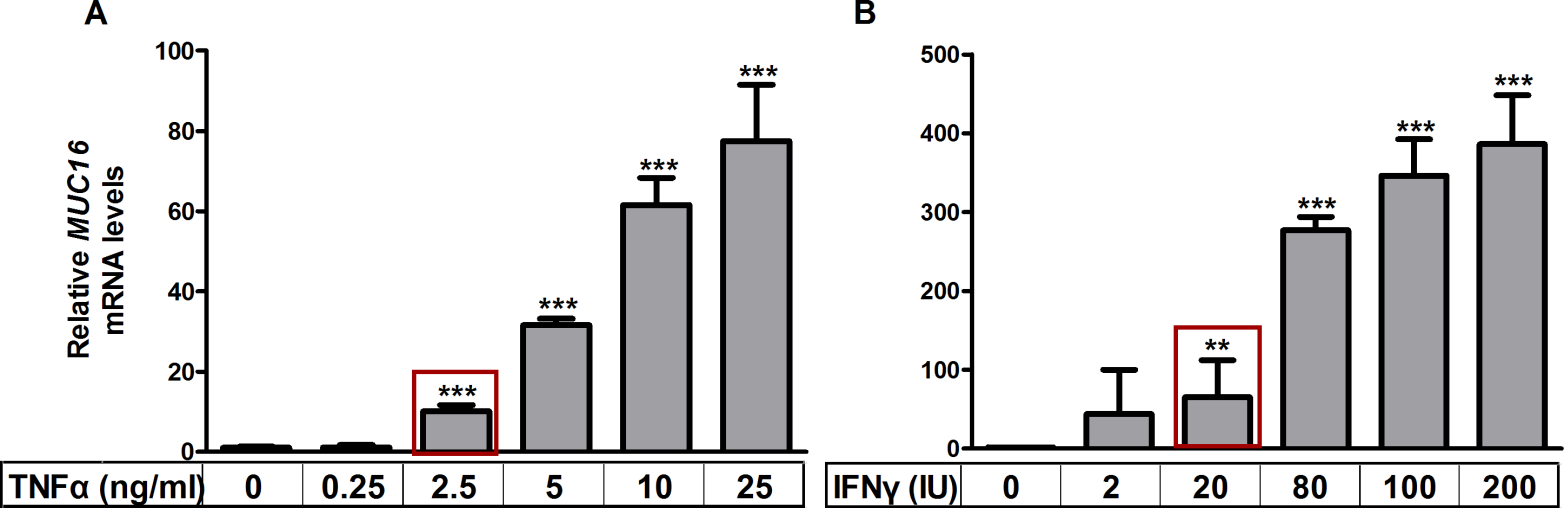
TNFα and IFNγ stimulate *MUC16* mRNA levels in a dose dependent manner MCF-7 cells were treated with the indicated concentrations of TNFα (panel **A**) or IFNγ (panel **B**) for 48 h, RNA extracted and the levels of *MUC16* mRNA relative to those of *ACTB* were determined by qRT-PCR as described in Materials and Methods. The boxes indicate the lowest concentrations of either cytokine that demonstrated a significant stimulation of *MUC16* mRNA levels and were used in subsequent experiments to determine the potential synergy of action between these cytokines. Values for the vehicle controls were arbitrarily set to 1 in each case. Bars and error bars indicate the means +/− SD for triplicate determinations in each case. **p < 0.01 *vs.* vehicle; ***p < 0.001 *vs*. vehicle.

Pro-inflammatory cytokines stimulate expression of MUC1 and MUC4 in other contexts [20], but little is known about MUC16 responsiveness in this regard. Initially, we determined the dose responsiveness of *MUC16* mRNA expression to either TNFα or IFNγ in MCF-7 cells, which contained the lowest basal levels of *MUC16* (Figure 1). TNFα was added at concentrations ranging from 0.25 ng/ml to 25 ng/ml for 48 h. IFNγ was added at concentrations of 2 IU to 200 IU for 48 h. In many experiments with MCF-7 cells, but not with other cells tested, extremely robust stimulation by cytokines was observed (> 50 fold); however, in other experiments stimulation was as low as 8-fold (Figure 4). Decreased responsiveness correlated to passage number and reflected a higher basal level of MUC16 expression with increasing passages. The lowest concentrations of either cytokine that demonstrated a significant stimulation of *MUC16* mRNA levels were 2.5 ng/ml of TNFα and 20 IU/ml of IFNγ (Figure 2A and 2B). Therefore, these concentrations were used in subsequent experiments to determine the potential synergy between these cytokines in stimulating MUC16 expression.

### Treatment with TNFα and IFNγ stimulated MUC16 mRNA levels in multiple cell types

**Figure 3:**
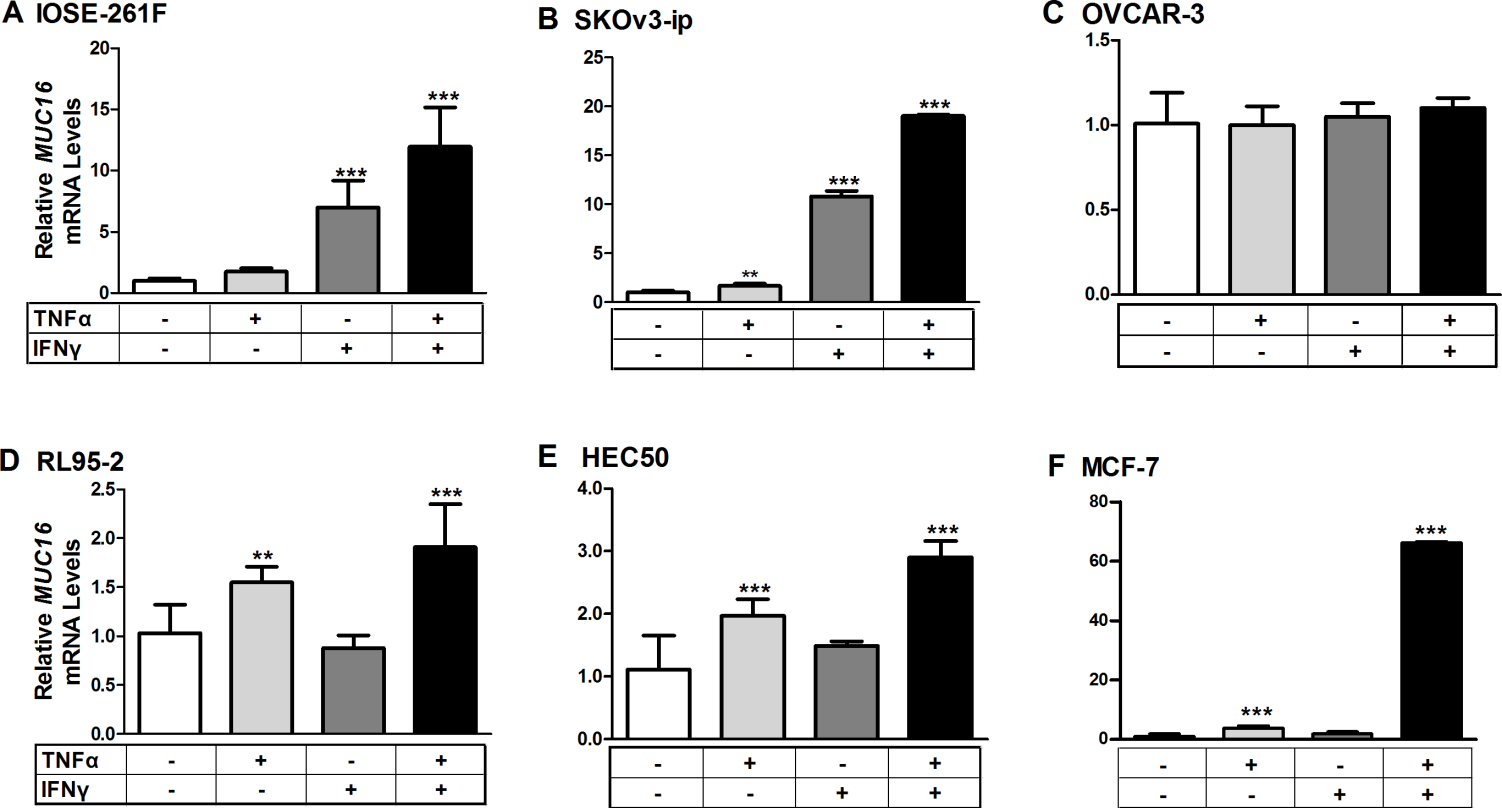
Cytokine treatments stimulate *MUC16* mRNA levels in various cellular contexts Each indicated cell line was treated for 48 h with either a vehicle control (0.1% [w/v] BSA in PBS), TNFα (2.5 ng/ml), IFNγ (20 IU), or a combination of TNFα + IFNγ. RNA then was extracted and the levels of *MUC16* mRNA relative to that of *ACTB* was determined by qRT-PCR as described in Materials and Methods. Values obtained for vehicle controls in each case were set to 1 for comparison. The bars and error bars indicate the mean ± SD of triplicate independent determinations in each case. ***p < 0.001, **p < 0.01 vehicle *vs.* IFNγ, TNFα or TNFα + IFNγ. Panels: (**A**) IOSE261F; (**B**) SKOv3-ip; (**C**) OVCAR-3; (**D**) RL95-2; (**E**) HEC50 and; (**F**) MCF-7.

The ability of low concentrations of TNFα (2.5 ng/ml) and IFNγ (20 IU) to stimulate *MUC16* mRNA expression was assessed in multiple cell types. Cells were treated for 48 h with either a vehicle control, or low cytokine doses individually or in combination (Figure 3A–3F). Values obtained for vehicle controls in each case were set to 1 for comparison. Treatments with the individual cytokines only modestly stimulated MUC16 gene expression (2–3-fold) in any cell context; however, combined cytokine treatment provided significantly higher stimulation in 5 of 6 cell types. The sole exception was OVCAR-3 cells, which display extremely high basal levels of *MUC16* mRNA (Figure 1). Higher cytokine concentrations in combination did not further stimulate *MUC16* expression in any context (data not shown). Therefore, the stimulation observed with the combined cytokine treatments appeared to be maximal in each case. The strongest stimulations were observed with cells displaying the lowest basal *MUC16* levels, namely IOSE 261F, SKOv3-ip and MCF-7 (Figure 3A, 3B and 3F). Modest, predominantly TNFα-dependent, stimulation was observed in the two uterine adenocarcinoma cell types, HEC50 and RL95-2 (Figure 3D and 3E), which displayed intermediate basal levels of *MUC16*.

### MUC16 protein expression and shedding were stimulated in response to treatment with a combination of TNFα and IFNγ

**Figure 4:**
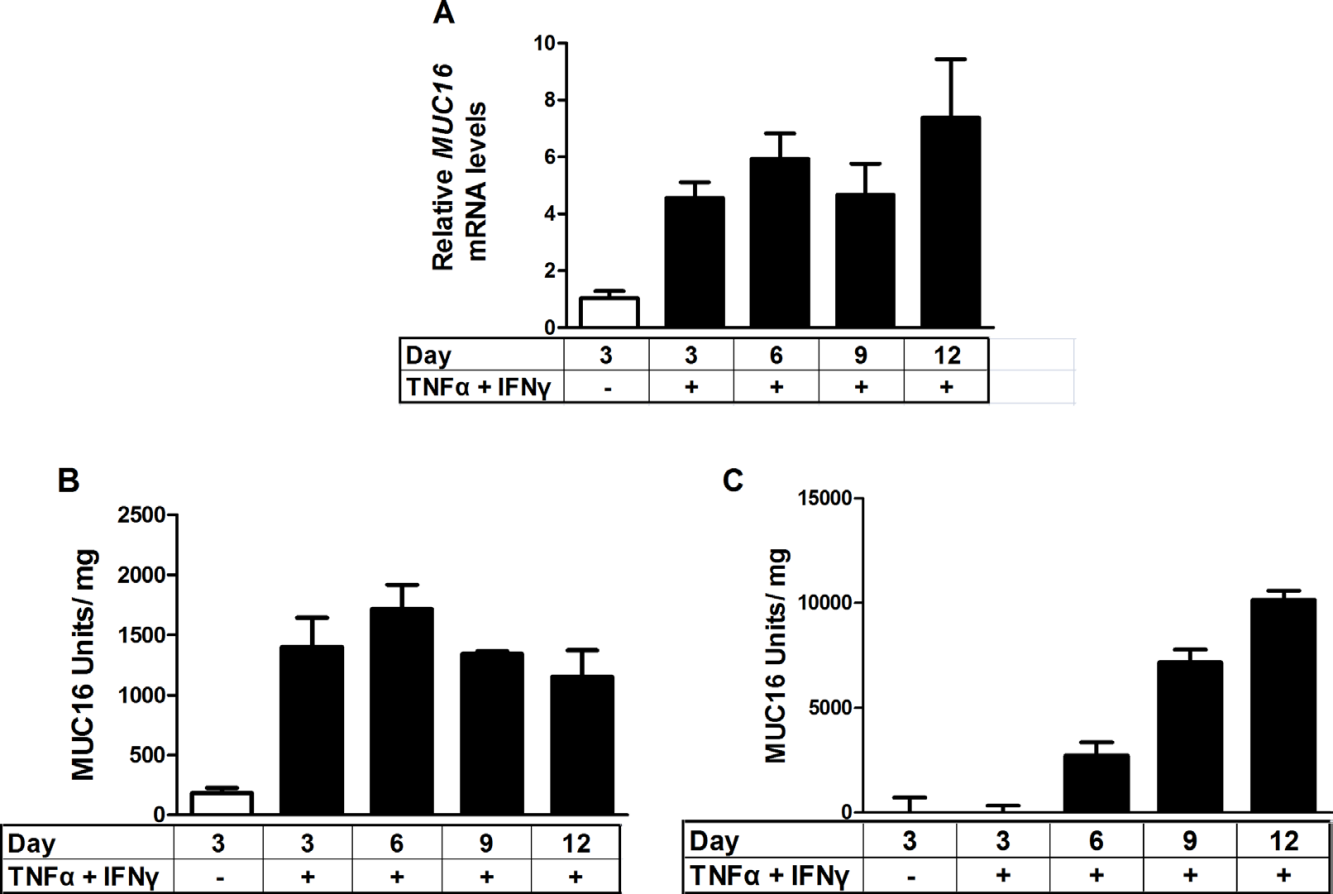
Time course of stimulation of *MUC16* mRNA levels and protein production in response to combined cytokine treatment MCF-7 cells were treated either with vehicle (0.1% [w/v] BSA in PBS) or TNFα (2.5 ng/ml) plus IFNγ (20 IU) for the indicated times. *MUC16* mRNA levels were determined by qRT-PCR and amounts of cell-associated and secreted/shed MUC16 were determined by CA 125 ELISA assays as described in Materials and Methods. The bars and error bars indicate the mean ± SD of triplicate independent determinations in each case. Panels: (**A**) mRNA levels; (**B**) cell associated MUC16; (**C**) media MUC16.

**Figure 5:**
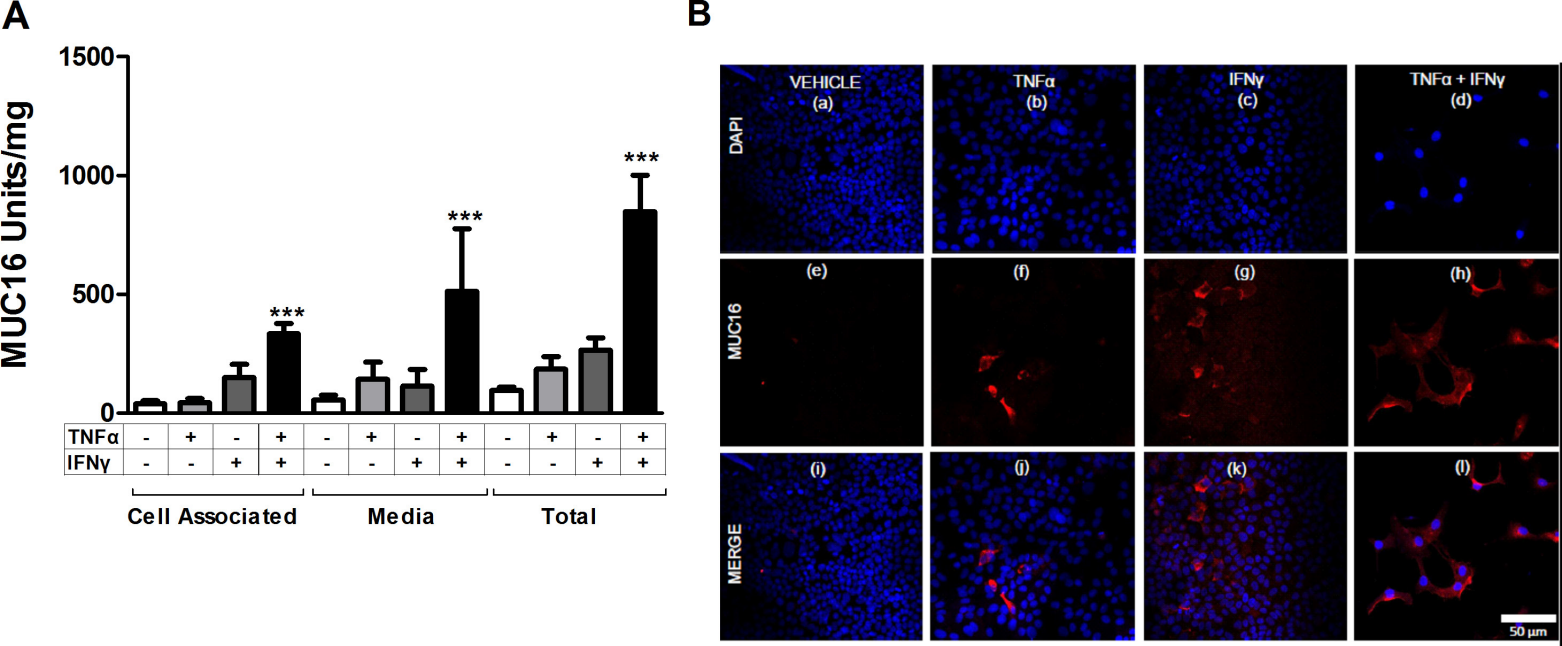
Individual cytokines stimulate MUC16 protein production in MCF-7 cells MCF-7 cells were treated for 6 days either with vehicle (0.1% [w/v] BSA in PBS), TNFα (2.5 ng/ml), IFNγ (20 IU), or TNFα + IFNγ. MUC16 levels then were determined (**A**) in the cell associated and media fractions by CA125 ELISA and (**B**) by immunofluorescence staining as described in Materials and Methods. Total MUC16 production was calculated by summing the amount of CA125 reactivity in the cell-associated and secreted fractions for each sample (**A**). A small stimulation of MUC16 production resulted from treatments with either TNFα or IFNγ alone. Much greater stimulation was observed with the combination of TNFα and IFNγ. The bars and error bars indicate the mean ± SD of triplicate independent determinations in each case. ***p < 0.001 vehicle *vs.* TNFα + IFNγ. (**B**) Vehicle; panels a, e and i; TNFα alone; panels **b**, **f** and **j**, IFNγ alone; panels c, g and k, or TNFα + IFNγ panels d, h and i. Cells were stained with MUC16 antibody (OC125; red) and DAPI (blue) and imaged. Prior to immunostaining, cells were treated for six days with vehicle (0.1% [w/v] BSA in PBS; Figure 5B panels **a**, **e** and **i**), TNFα (2.5 ng/ml; Figure 5B panels **b**, **f** and **j**), IFNγ (20 IU; Figure 5B panels **c**, **g** and k), or TNFα + IFNγ (Figure 5B panels **d**, **h** and **l**). Cells subsequently were fixed and stained with MUC16 antibody (OC125; red) and DAPI (blue) and imaged.

Because MCF-7 cells displayed the most robust response to cytokine treatment, they were used to perform time course studies for MUC16 (CA125) protein and mRNA accumulation. MUC16 mRNA (Figure 4A) and cell-associated protein (Figure 4B) levels were essentially maximal within 6 days of cytokine exposure while media levels continued to accumulate throughout the 12 day time course (Figure 4C). It also was found that for MCF-7 cells about 90% of the MUC16 protein produced was ultimately found in the media by 12 days of treatment (Figure 4C). Consequently, the cytokine stimulation of *MUC16* mRNA expression was also reflected at the level of MUC16 protein expression. We used six days of treatment to examine the actions of individual cytokines on MUC16 protein expression. A small increase in MUC16 stimulation in the cell-associated fraction resulted from treatment with IFNγ alone (*p < 0.05). Nonetheless, much greater stimulation was observed with TNFα or TNFα plus IFNγ in the media and total fractions indicating that TNFα was a strong driver of MUC16 production (***p < 0.001 vehicle vs TNFα + IFNγ) (Figure 5A).

The effect of cytokine treatment on cell surface MUC16 was investigated further in MCF-7 cells by immunostaining (Figure 5B panels a through l). The promotion of MUC16 expression was a consistent response in 5 of the 6 cell types tested.

### MUC16, TNFα and IFNγ are coexpressed in malignant uterine neoplasms

**Figure 6:**
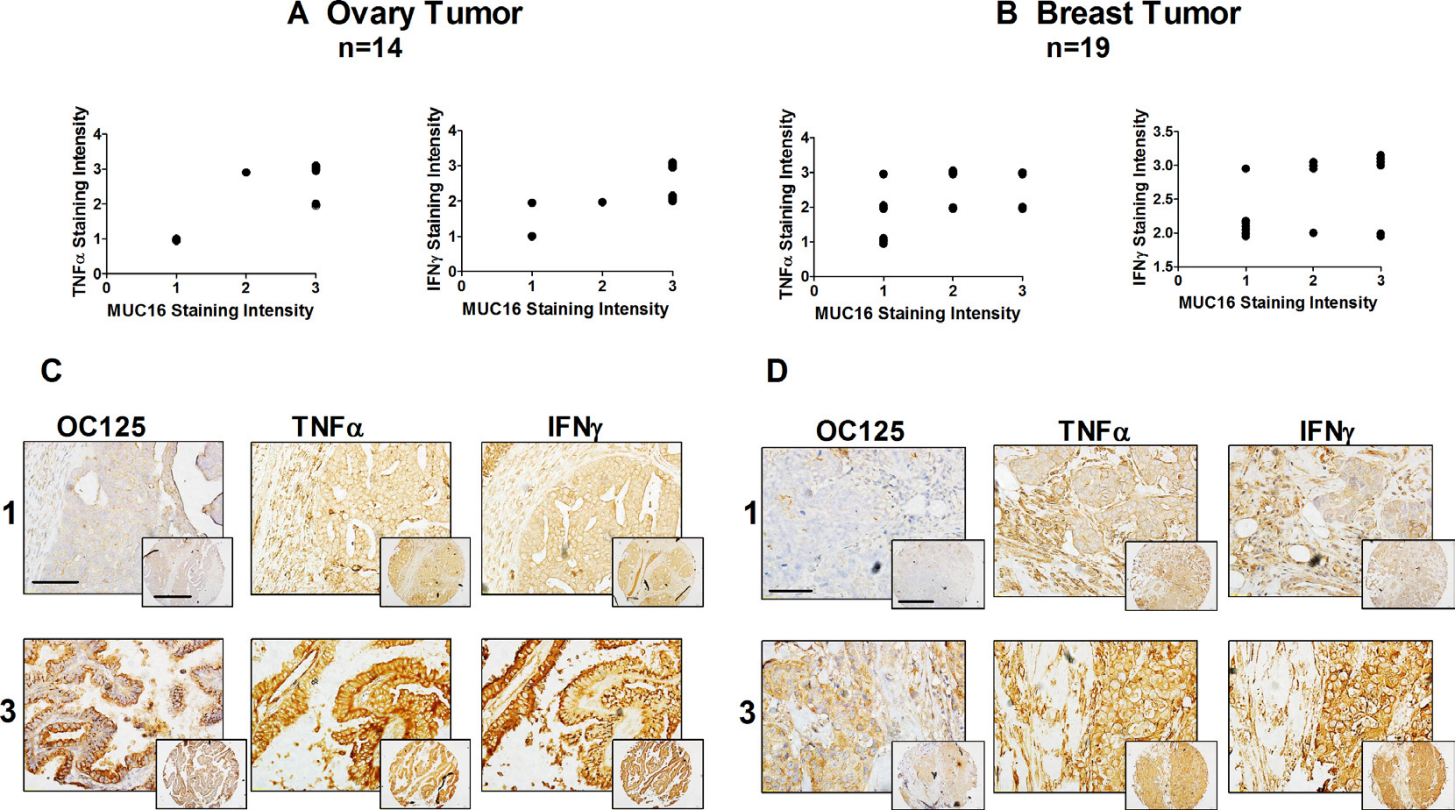
MUC16, TNFα, and IFNγ are co-expressed in malignant ovarian and uterine neoplasms Tissue staining and staining intensity assessments were performed as described in Materials and Methods. A correlation between the intensity of MUC16 staining and TNFα and IFNγ staining was observed in ovarian cancer (**A**) and to a lesser degree in breast cancer (**B**). Cytokine staining intensity was plotted against the staining intensity for MUC16 in each case and a correlation analysis was performed. Ovarian cancer TNFα *vs* MUC16: Correlation coefficient (r) = 0.8016. r squared = 0.6426, The *P* value is 0.0006, considered extremely significant and IFNγ *vs* MUC16: Correlation coefficient (r) = 0.7077. r squared = 0.5009, the *P* value is 0.0046, considered very significant. Breast cancer, TNFα *vs* MUC16: Correlation coefficient (r) = 0.4771. r squared = 0.2276, the *P* value is 0.0389, considered significant and IFNγ *vs* MUC16: Correlation coefficient (r) = 0.7705. r squared = 0.593, the *P* value is 0.0001, considered extremely significant. Data was plotted with a small variation to help visualization. Representative tissue microarray images show different combinations of MUC16, TNFα and IFNγ expression in different uterine neoplasms. (**C**) Representative staining of ovarian cancers scored as 1 or 3 for MUC16, TNFα and IFNγ with staining in both the cancer and stroma. Lower magnification, scale bars: 25 μm. Higher magnification (inset), scale bars: 10 μm. (**D**) Representative staining of breast cancers scored as 1 or 3 for MUC16, TNFα and IFNγ. Lower magnification, scale bars: 25 μm. Higher magnification (inset), scale bars: 10 μm.

The *in vivo* association of cytokine expression with that of MUC16 was assessed with immunohistochemical staining of a multi-tumor human tissue microarrays. Serial sections of a human cancer tissue array were stained simultaneously using anti-MUC16 or anti-TNFα or anti-IFNγ antibody. The array included various cancers including ovary, endometrium and breast, which were the focus of this study (Supplementary Table 1). The purpose of these studies was to determine if relative MUC16 expression correlated with TNFα or IFNγ expression, independently of tumor stage or grade. Samples were classified according to staining intensities, which represents protein expression. The array staining generally revealed that strong cytokine expression was associated with elevated MUC16 expression in many cancers. In cancers such as ovarian (Figure 6A and 6C) and breast (Figure 6B and 6D), there was a direct correlation between the staining intensity for both cytokines and MUC16. The correlation for endometrial cancer was not as clear (graph not shown).

### MUC16 mRNA responses to cytokines are attenuated when NFκB/p65 is knocked down by siRNA

**Figure 7:**
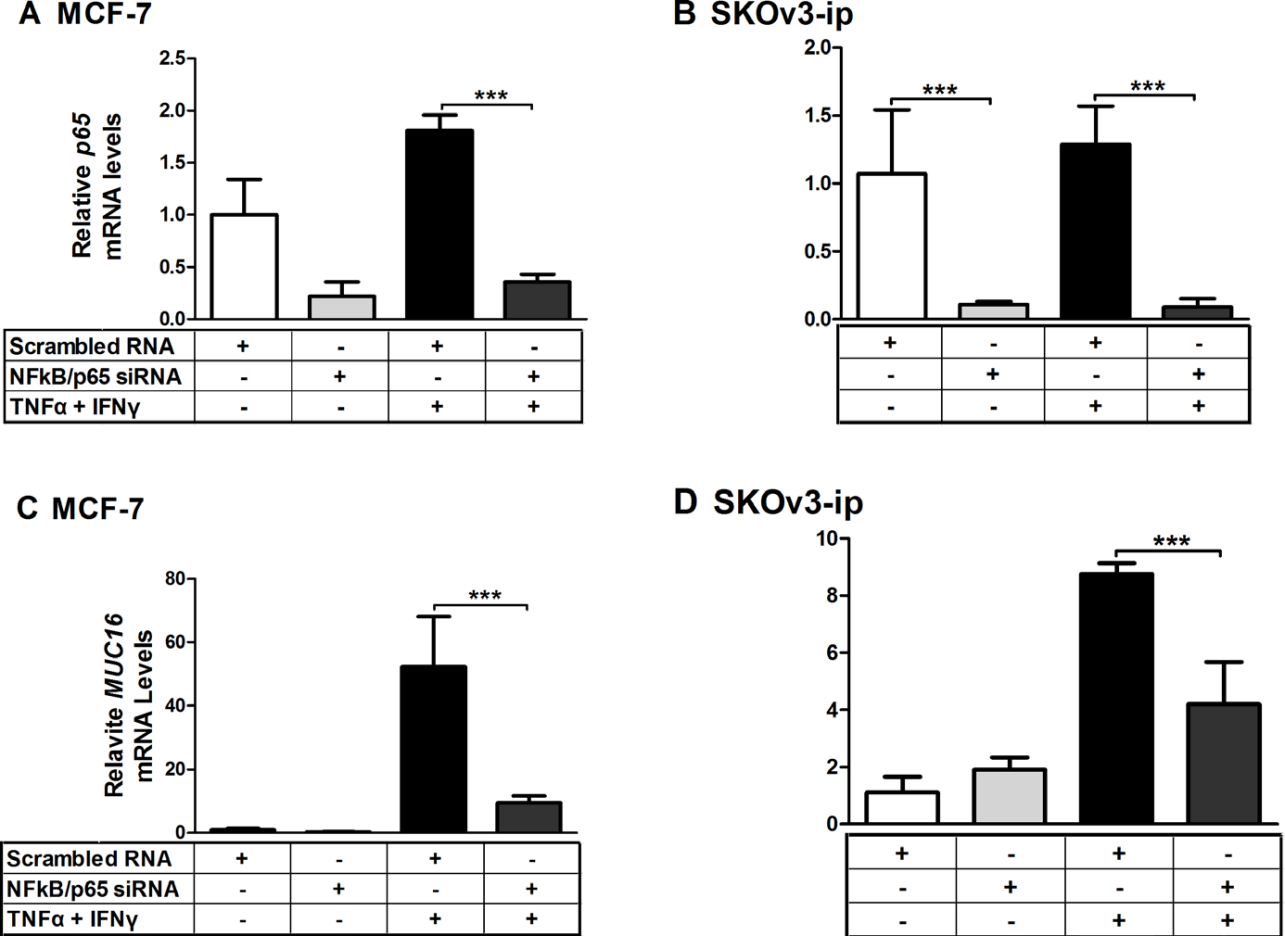
*MUC16* mRNA responsiveness to cytokines is attenuated when NFκB/p65 is knocked down by siRNA MCF-7 (**A**, **C**) and SKOv3-ip (**B**, **D**) cells were treated for 24 h with either scrambled or NFκB siRNA at a final concentration of 50 nM. Cells then were treated for 24 h with either a vehicle control (0.1% [w/v] BSA in PBS), or a combination of TNFα (2.5 ng/ml) plus IFNγ (20 IU). RNA then was extracted and the levels of NFκB/p65 (**A** and **B**) and *MUC16* (**C** and **D**) mRNA relative to that of *ACTB* were determined by qRT-PCR as described in Materials and Methods. Values obtained for scrambled vehicle control in each case were set to 1 for comparison. The bars and error bars indicate the mean ± SD of triplicate independent determinations in each case. ***p < 0.001.

Given the particularly strong actions of TNFα on MUC16 expression, we sought to determine if the key downstream transcription factor, *NFκ*B, mediated this response. To accomplish this, NFκB/p65 was knocked down *via* siRNA in two cell types displaying the strongest *MUC16* mRNA elevation in response to cytokines, namely MCF7 and SKOv3-ip. NFκB/p65 knockdown reduced target mRNA and protein levels by 60–80% of the control (Figure 7A and 7B and Supplementary Figure 1). Cytokine stimulated *MUC16* mRNA levels were significantly (*p* < 0.001) decreased when NFκB/p65 was knocked down in both cell types (Figure 7C and 7D). This also was the case even when TNFα or IFNγ were added individually (Supplementary Figure 2). Collectively, these studies demonstrate that NFκB is an important mediator for both TNFα and IFNγ responsiveness. While was not further elevated *MUC16* expression in OVCAR-3 cells in response to cytokines (Figure 3), we used siRNA-mediated knockdown to determine if NFκB mediated high level *MUC16* expression in this cell context. Though we again achieved effective p65 knockdown (> 80%), no changes in MUC16 mRNA levels were noted (data not shown). Thus, the extremely high basal levels of *MUC16* in this cell line appear to be due to processes independent of NFκB.

### A conserved, consensus, NFκB binding site in the proximal MUC16 promoter accounts for much of the cytokine responsiveness

**Figure 8:**
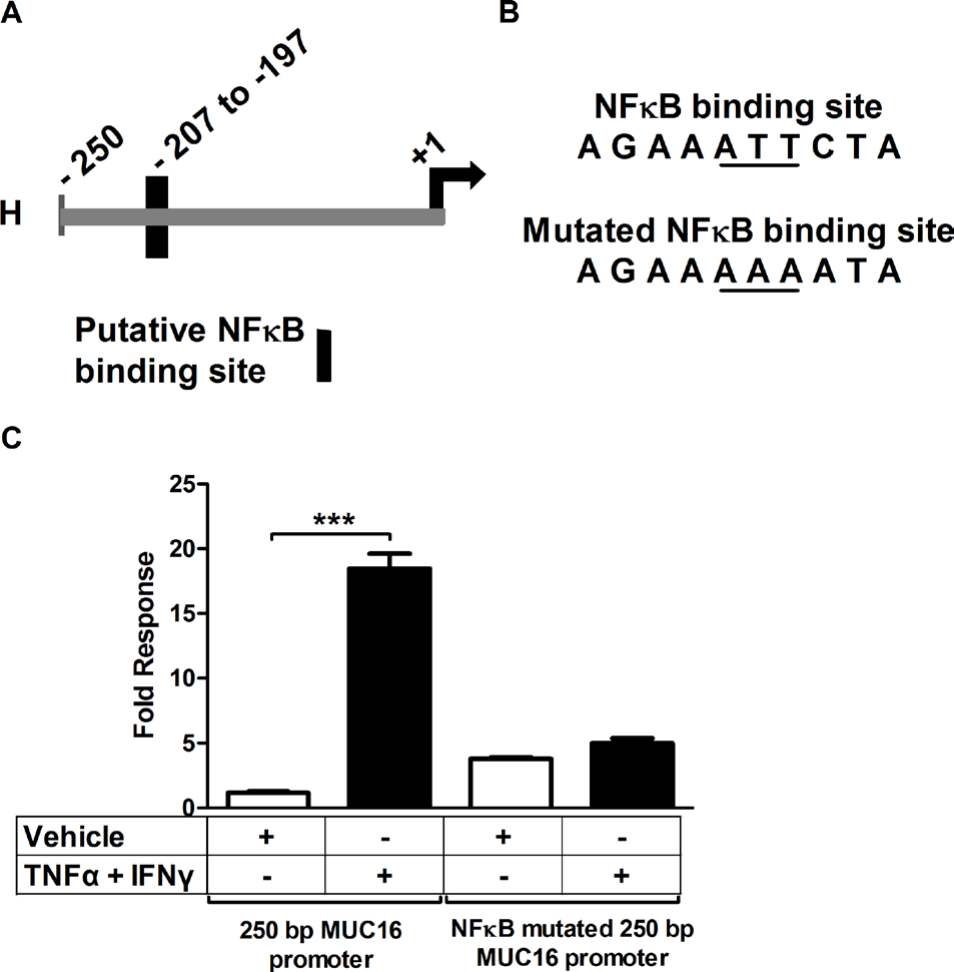
A consensus NFκB-binding site accounts for much of the MUC16 gene responsiveness to cytokines (**A**) Diagram of the proximal 250 bp *MUC16* promoter indicating the position of the putative NFκB binding site. +1 refers to the 5′RACE determined start of transcription for MCF-7 cells. (**B**) Sequence of the potential NFκB binding site and the mutated site generated for the transfection studies described in C. (**C**) MCF-7 cells were transfected with the wild type 250 bp proximal *MUC16* reporter plasmid or the mutated putative NFκB binding site and then treated with either vehicle (0.1% [w/v] BSA in PBS), or TNFα (2.5 ng/ml) + IFNγ (20 IU) for 24 h before measuring luciferase activity in cell lysates cells as described in Materials and Methods. Data are reported as the values obtained relative to the vehicle-treated wild type promoter. The bars and error bars indicate the mean ± SD of triplicate independent determinations in each case. ***p < 0.001 wild type promoter vehicle *vs.* TNFα + IFNγ.

Knowing that the cytokine response was associated with the transcription factor NFκB, we next studied the MUC16 promoter to look for binding sites for NFκB and associated transcription factors. The start of transcription of the MUC16 gene has been predicted, but until now has not being biochemically determined. Therefore, we performed 5′ RACE to determine the start site of MUC16 transcription. Two cell types were used for 5′ RACE, MCF-7 and OVCAR-3. It was found that *MUC16* has alternative sites of transcription; it is cell type specific and differs by a few nucleotides from the predicted start site (Supplementary Figure 3). We next performed a bioinformatics analysis of the region 2000 bp upstream of the transcriptional start site to determine if any consensus NFκB binding sites occurred in this region and if they were conserved between mice and humans. In the region within 200 bp from the start of transcription (Figure 8A) a consensus NFκB binding site was found in addition to others such as AP-1 and Sp1. To determine if the NFκB binding site was associated with the cytokine response, we cloned the proximal 250 bp of the *MUC16* promoter into a pGL3 basic luciferase vector. Transient transfection reporter assays revealed that the 250 bp promoter accounts for a large portion of the cytokine responsiveness. Furthermore, mutating the NFκB binding site destroyed the cytokine response (Figure 8B and 8C). Thus, in addition to identifying NFκB as a key transcription factor mediating cytokine responsiveness of the *MUC16* gene, we also identified a key consensus NFκB binding element in the MUC16 promoter responsible for cytokine responsiveness.

### Binding and recruitment of NFκB/p65 to the MUC16 promoter *in vitro* and *in vivo*

To determine if NFκB directly interacts with the consensus NFκB, both gel shift (EMSA) and chromatin immunoprecipitation (ChIP) assays were performed. Oligonucleotides either containing (EMSA) or flanking (ChIP) the putative NFκB binding site were designed for both assays, as well as an additional oligonucleotide containing a mutated NFκB for the EMSA assay as described in Material and Methods. As expected, the wild type oligonucleotide interacted with the recombinant NFκB/p65 protein, while the mutated site showed very weak binding (Supplementary Figure 4A). ChIP experiments also showed an increased occupancy of the MUC16 promoters by NFκB following cytokine treatment when compared with the untreated control (Supplementary Figure 4B)

## DISCUSSION

In the current study we demonstrated that the pro-inflammatory cytokines TNFα and IFNγ stimulate MUC16 expression in a variety of cellular contexts. Furthermore, we showed that elevated MUC16 expression is associated with elevated cytokine levels in several cancers of female reproductive tissues. We identified NFκB and a key NFκB binding site in the proximal *MUC16* promoter as key mediators of this response.

Stimulation of MUC16 expression by cytokines has been reported in ocular surface epithelial cell lines [24, 25]. In the present study we found that cytokines increase MUC16 mRNA and protein levels in a variety of epithelial cancer cell types. Knowing that cytokines stimulate expression of multiple transmembrane mucins suggests a general response potentially intended to enhance the protective functions of epithelia [20, 28]. In general, the degree to which cytokines stimulated MUC16 expression was related to the basal level of MUC16 expression, i.e. the lower the basal MUC16 level was the greater stimulation observed with cytokines. We found that maximal MUC16 expression was observed with very low concentrations of cytokines used in combination. This suggests that *in vivo* low cytokine levels diffusing throughout a tumor may strongly potentiate MUC16 expression.

While a large number of studies report serum cytokine levels or increases in cytokine mRNA levels in tumor tissues, very few have measured cytokine levels in tumor tissues [29]. These studies generally indicate that levels of TNFα and IFNγ are low in these tissues, but in the range where we observed cooperative stimulation of MUC16 expression. It is likely that cytokine concentration gradients are created, radiating from the cellular sources of their production. Nonetheless, the ability of these cytokines to cooperatively elevate MUC16 indicates that even low amounts of cytokines are sufficient to drive this response in tumors. Moreover, these gradients may also account for heterogeneity in MUC16 expression within tumor tissues.

Cytokine stimulation of mucin expression in cancer cells likely reflects conservation of a biological response of normal mucosal epithelia. Cytokines are elevated in normal tissues in response to injury or infection to prompt the immune system to respond to the challenge. As part of this response, it appears that mucins are overproduced to protect the site from further damage or pathogenic invasion. In the case of cancer, these responses protect the mucin-expressing cancer cells from the immune system [17, 30] and cytotoxic drug penetration [30, 31]. MUC16 can also carry ligands for immuno-inhibitory Siglecs [32, 33]. Thereby, the mucin response to cytokines represents a “vicious cycle”. Overexpression and shedding of MUC16 is predicted to contribute to immunosuppressive activities further protecting tumor cells from host immune responses.

While many normal epithelial cells express MUC16 [25, 34–36], it is not clear if shedding or cellular retention is the major metabolic fate. We found that ∼66% of the MUC16 produced by MCF-7 cells is released to the medium. The mechanism for this release is unclear at present, but may involve cell surface proteases as is the case for other transmembrane mucins [37, 38]. This is the first study describing the distribution of MUC16 between the cell-associated and shed/secreted compartments. More work detailing the mechanism of release as well as whether this occurs in other cell types is needed to know if this reflects a general behavior of MUC16 or is specialized to particular cell types. In addition, determination of the biological functions of shed MUC16, beyond serving as a valuable serum marker, is needed. The energy expenditure in producing this huge glycoprotein (ca. 22,000 amino acids) heavily decorated with > 1 × 10^6^ sugar residues is extraordinary. An estimate of 2 nucleoside triphosphates (NTPs) used per amino acid addition and 36 ATPs potentially generated by complete oxidative metabolism of each hexose indicates an energetic cost of > 36 ×−10^6^ NTPs to produce one MUC16 molecule. Given this tremendous metabolic investment, it seems likely that the functions associated with MUC16 and its shed fragments are important. Released MUC16 binds to the surfaces of various immune cells consistent with the aforementioned suggestion of an immunosuppressive function [11, 39]. Nonetheless, Muc16 null mice display no overt phenotypes [40]. It is possible that MUC16 functions in these mice are not manifest until presented with an appropriate challenge. MUC16 also binds the cell surface protein mesothelin on mesothelial cells, perhaps providing a way for migrating MUC16-expressing cells to colonize ectopic sites, e.g. the peritoneum.

In this study we found that TNFα, IFNγ and MUC16 are often strongly co-expressed in endometrial, breast and ovarian cancers with cytokine staining intensity positively correlating with MUC16 staining in breast and ovarian cancers, in particular. These data are consistent with the notion that elevated cytokines in the tumor microenvironment induce the production of MUC16. We suggest that elevated MUC16, in turn, protects the tumor against the immune system and cytotoxic drug penetration.

Our data provides important, novel information on cytokines as factors driving MUC16 expression in cancer cells and possibly in normal tissues as well. Regulation of MUC16 expression through NFκB could aid in early detection of ovarian cancer. CA125 (MUC16) levels can rise months to years prior to conventional diagnosis of ovarian cancer [41]. While administration of TNFα of IFNγ may or may not be tolerable clinically, selective enhancement of NFκB and MUC16 levels in cancer cells by other methods might provide a provocative test to confirm the presence of early stage ovarian cancer in asymptomatic women with rising levels of serum CA125 (MUC16). As MUC16 is likely important for metastasis in the peritoneal cavity [42], anti-inflammatory strategies might slow progression of the disease. Interfering with cytokine actions in tumor cells then represents an avenue to develop therapeutic approaches to reduce MUC16 levels to increase cancer cell susceptibility to the host immune system and cytotoxic drugs.

## MATERIALS AND METHODS

### Cell culture

Well-established cells with known genomic characterization were used. RL95-2 and HEC50, were cultured in DMEM/Hams F12 (Life Technologies; 11330–057). IOSE 261F were cultured in MCDB 106 (SIGMA)/MEDIUM 194 (MEDIATECH) [1:1, v/v]. SKOv3-ip, were cultured in McCoy's 5A (Thermo Scientific; SH30270.01). OVCAR-3, were cultured in RPMI 1640 (Gibco^®^; 11875119). MCF-7 (last profiled on 7/22/13 by STR profiling at Johns Hopkins Genetic Resources Core Facility), were cultured in Minimum Essential Medium (MEM) (Life Technologies; 11095098). All media were supplemented with 10% (v/v) fetal bovine serum (FBS) and penicillin (100 U/ml)–streptomycin (100 μg/ml) (Gibco^®^; 15140–122). MCF-7 and OVCAR-3 cell media also were supplemented with 10 μg/ml of insulin (v/v) (Sigma-Aldrich Inc.; I9278).

### Cytokine treatments

Cells were seeded in six well plates in media containing 5% (v/v) charcoal-stripped FBS (Life Technologies; 12676029) and allowed to reach 60–70% confluence. Cells then were gently rinsed with PBS and then serum free medium was added for 24 h. Cells then were treated with tumor necrosis factor alpha (TNFα, ROCHE; 113718430) and or interferon gamma (IFNγ ROCHE; 11040596001) at the concentrations indicated in the text in media containing 10% (v/v) charcoal-stripped FBS. Cells were incubated with cytokines for 48 h prior to RNA extraction and for six days prior to protein extraction and immunostaining, and for up to 12 days in time course experiments.

RNA isolation and reverse transcription-PCR. Total RNA was isolated by using TRIZOL reagent (Invitrogen; 15596–026) and chloroform (Cambridge Isotope Laboratories; DLM-7TB-100). Samples then were treated with DNAse according to the manufacturer's instructions (Ambion; AM1906). Reverse transcription was performed using 1 μg of total RNA in a 10 μl reaction using qScript cDNA Super mix (Quanta; 95048) incubated for 5 min at 25°C, 30 min at 42°C and 5 min at 85°C. Real time qPCR was performed using the following primer sequences: MUC16 forward, 5′-GCC TCTACCTTAACGGTTACAATGAA-3′ and reverse, 5′-G GTACCCCATGGCTGTTGTG-3′ [25] beta actin (ACTB) forward, 5′-GATGAGATTGGCATGGCTTT-3′ and reverse, 5′-CACCTTCACCGGTCCAGTTT-3′ [43] and NFκβ forward, 5′-ATCTGCCGAGTGAACCGAAACT-3′ and reverse, 5′-CCAGCCTGGTCCCGTGAAA-3′ [44] and SYBR Green Super mix according to the manufacturer's instructions (Quanta Bioscience). Samples were cycled as follows: *MUC16*, (30 sec at 95°C and 30 sec at 59°C for 40 cycles); *NFκB p65* (an initial incubation at 50°C for 2 min followed by 40 cycles of 95°C for 10 min, 95°C for 15 s and 60°C for 1 min [44]). Relative amounts of mRNA were identified using the comparative threshold cycle method [45, 46] and normalized to that of *ACTB*.

### Immunofluorescence analysis

Cells (5 × 10^4^) were plated in 8-well chamber slides and treated with cytokines for six days. Cells were washed with PBS and fixed with 100% methanol for 10 min at room temperature. Subsequently, cells were washed and allowed to re-hydrate for 10 min at room temperature in PBS. Slides were washed three times with PBS, blocked for 1 h with [3% (w/v) BSA in PBS] and washed three times with PBS. Primary antibody was added and incubated overnight at room temperature at the indicated dilutions: mouse monoclonal anti-MUC16 (OC125); 1:100 in blocking solution. Slides were washed three times with PBS, then incubated with 1:400 dilution of Alexa-fluor 647 goat anti-mouse (Invitrogen; A21235) in blocking solution at room temperature for 1 h in the dark, and washed three times for 5 min at room temperature with PBS. Samples were mounted with Prolong-Antifade with 4′, 6-diamidino-2-phenylindole (DAPI), a fluorescent molecule that binds strongly to A-T regions in DNA in the nucleus, as per manufacturer's instructions (Life Technologies; P-36931) and viewed by confocal microscopy on a Zeiss LSM710 microscope.

### ELISAs

Cells were plated (4 × 10^5^) in six-well plates and treated with cytokines for six or up to 12 days. Fresh media with cytokines was changed every three days and conditioned media was saved. Protein from cell lysates was isolated using 500 μl of RIPA lysis buffer (Santa Cruz; sc-24948). ELISA assays were performed using CA125 ELISA kit (BQ kits; BQ1013T) following the manufacturer's instructions.

### siRNA knockdown

NFκB was knocked down using an oligonucleotide targeting p65 (Santa Cruz; sc-29410/human NFκB p65 siRNA) and a non-silencing, scrambled siRNA control: (5′-AATTCTCCGAACGTGTCACGT-3′ [47]. All siRNAs were resuspended in RNAse-free water to a final concentration of 10 μM by adding 330 μl of RNase-free water to 3.3 nmol of lyophilized siRNA for NFκB p65 and by adding 1 mL of RNAse-free water to the lyophilized siRNA to achieve a final concentration of 20 μM for the scrambled control. Oligonucleotides were transfected using Lipofectamine 2000 (Invitrogen; 11668019) according to the manufacturer's instructions. Briefly, cells were plated in antibiotic free media in 12 well plates incubated for 24 h at 37° in a humidified atmosphere of air: CO_2_ (95:5, v/v). Oligonucleotides were transfected at a final concentration of 50 nM in Opti-MEM^®^ Reduced Serum Medium, GlutaMAX^™^ Supplement (Gibco; 51985034). Six h after transfection, media was changed to regular medium plus FBS lacking antibiotics. Twenty four h later cells were treated with or without cytokines for 24 h followed by RNA extraction.

### Immunohistochemistry

Formalin-fixed, paraffin-embedded tumors were resected from 94 patients and obtained from the archives of Department of Pathology, MD Anderson Cancer Center (Houston, TX). The MDACC Institutional Review Board approved the use of tissue. To provide a positive control, SKOv3-ip cells were grown for 24 h in McCoy's medium supplemented with 10% (v/v) FBS, 1% (penicillin [100 U/ml]–streptomycin [100 μg/ml], and 1% [w/v] L-glutamine. Cells were harvested in 0.25% (w/v) trypsin (Fisher Scientific, MT-25-052-CI) washed 2 times in PBS, fixed in 10% (w/v) formalin and embedded in paraffin. Oven incubation at 60°C for 20 min was used to deparaffinize slides followed by two 20 min incubations at room temperature in xylene. After slides were rehydrated, antigen retrieval was performed in 6.5 mM sodium citrate buffer (pH 6.0) for 10 min. Blocking was performed with 5% (w/v) bovine serum albumin in PBS. The primary antibody (anti-TNFα mouse monoclonal, 1:200, EDM Millipore MAB1096; anti-IFNγ rabbit polyclonal, 1:250, Novus Biologicals NBP1-19761; or OC125 mouse monoclonal 1:400, R.C. Bast Laboratory) were incubated at 4°C overnight. Mouse or rabbit secondary antibody (Jackson ImmunoResearch) was applied for 1 h at room temperature followed by washing 3 times in PBS for 10 min. Diaminobenzidene chromagen (Biocare Medical, BDB 2004L) was added for 1 min per slide followed by 3 additional washes in PBS for 10 min and then hematoxylin staining was performed for 1 min per slide followed by 3 additional washes in PBS for 10 min. Serial sections of paraffin-embeded OVCAR-3 cells and non-immune IgG staining served as positive and negative controls, respectively, and were stained alongside tissue microarray (TMAs) to confirm assay reproducibility. Omission of the primary antibody served as an additional negative control for each immunostaining event. Immunohistochemical staining was evaluated for both overall staining intensity and location of the staining in the tumor or stroma alone versus diffuse staining in both tissues. Total staining intensity was determined as 0 (no staining), 1 (weak staining), 2 (moderate staining), and 3 (strong staining). All slides were evaluated independently by 2 investigators (ZL and MNS) without knowledge of the identity of the patient or clinical outcome.

### 5′ Rapid amplification of cDNA ends (RACE)

RACE PCR was performed to formally determine the *MUC16* transcriptional start site. These reactions were performed using the 5′RACE System (Life Technologies; 18374–058) according to the manufacturer's instructions. Primers used were, GSP1: 5′-CACCACGAT TGCACCTGTAG-3′ and GSP2 5′-TTAGTGCTCCTGC TCCCTGT-3′. The PCR products then were sequenced using the GSP3 primer 5′-CCAGAGGCAA ATGTTGACC T-3′.

### MUC16 promoter plasmid construction

Genomic DNA was purified from MCF-7 cells using a Wizard^®^ SV Genomic DNA Purification System (Promega: A2360). A construct containing 250 bp upstream of the start site of *MUC16* transcription (hereafter refer to as 250 bp promoter construct) was amplified by genomic PCR. This PCR product was cloned into pCR 2.1 TOPO (Life Technologies; K456001). This fragment was ligated into the promoter less pGL3 firefly luciferase reporter vector (Promega; E1751). The primers used were Fwd: 5′-AGAGAGAGAGAGAGAGAGGATCATT-3′ Rev 5′-AATGATCCTCTCTCTCTCTCTCTCT-3′. Site directed mutagenesis was performed using the QuikChange Lightning Site-Directed Mutagenesis Kit (Agilent Technologies Inc; 210518) according to the manufacturer's instructions.

### Transient transfection and reporter assays

MCF-7 or SKOv3-ip cells were plated in 5% (v/v) charcoal-stripped FBS in six-well plates and maintained for 48 h until the cells reached 80 to 90% confluence judged by eye. Transient transfections were performed using Lipofectamine 2000 (Invitrogen; 11668–019) and Opti-MEM (Life Technologies; 51985034) according to the manufacturer's instruction. Expression and reporter plasmids were added at 500 ng and 1 μg per well, respectively, and 10 ng of pRL-TK plasmid was used per well. After transfection cells were allowed to recover in serum free media for 12 h. Cytokine treatments were added as described above for 24 h. The Dual-Luciferase Assay kit (Promega; E1960) was used to lyse the cells and perform the luciferase assay according to the manufacturer's instructions. Reporter activity was expressed as the ratio of firefly luciferase activity to *Renilla* luciferase activity.

### Electrophoretic mobility shift assay (EMSA)

Recombinant NFκB p65 protein (Active Motif; 31102)(500 ng) was incubated with 40 ng of oligonucleotide probes for NFκB p65 containing either a consensus (5′-CGACATGATACACTA AGAAATTC TATTCTGCAGACACTGC-3′) or a mutated (5′-CGAC ATGATACACTAAGAAAAAATATTCTGCAGACACTG C-3′) sequence, similar to the NFκB sequence found in the MUC16 promoter by bioinformatics analysis and used in the luciferase assay, in a reaction mixture following the manufacturers instructions containing 2 μl of EMSA/Gel-shift binding buffer 5× from EMSA Kit (ThermoFisher; E33075) containing 750 mM KCl, 0.5 mM dithiothreitol, 0.5 mM EDTA, 50 mM Tris, pH 7.4 for 40 min. The reaction mixtures were separated by 12% non-denaturing PAGE. The gel was then stained for 20 min with 1× SYBR^®^ green staining solution; the gel was then washed with dH_2_0 two times for ∼2 sec followed by visualization using a Carestream Imaging system.

### Chromatin immunoprecipitation (ChIP)

The Chromatrap^®^ kit (Porvair Sciences – Leatherhead, Surrey, England) was used for ChIP assays. MCF-7 cells were grown to 60 to 80% confluency and serum-withdrawn overnight, then treated for 4 h with TNF-α (2.5 ng/mL) and IFNγ (20 IU) or vehicle control prior to collection of chromatin. Fixation, DNA shearing and ChIP were performed following the manufacturer's instructions. Antibodies included the anti-p65 SC-109X antibody (Santa-Cruz), as well as a control IgG antibody. Primer sequences used to amplify NFκB regulatory region are 1) Fwd: 5′-AGCCTGGTTCCTGGTTTCTAA-3′ and Rev: 5′-CCCTTCAAACTTTTTAACGGATT-3′ and 2) 5′-GCCTGGTTCCTGGTTTCTAA-3′.

Rev: 5′-TGATCTCAATTCTTCCCTTCAAA-3′. The *NFKBIA* gene promoter served as a positive control for p65 immunoprecipitation, and the GAPDH promoter was used a positive control for RNA polymerase II immunoprecipitation. The *HBB* gene was the negative control for nonspecific pulldown by either the antibody or adsorption by the column beads.

### Statistical analysis

All data is shown as the mean ± SD of triplicate determinations of independent biological samples. Statistical analyses were performed using a one-way ANOVA followed by Tukeys post-test using GraphPad InStat software, version 3.05 (GraphPad Software, San Diego, CA). Differences were considered significant when *P* < 0.05, two-tailed test.

## SUPPLEMENTARY MATERIALS TABLE AND FIGURES


