# Supplementary material for: Tumor necrosis factor-α and interferon-γ stimulate MUC16 (CA125) expression in breast, endometrial and ovarian cancers through NFκB
Source: Oncotarget. 2016 Feb 24;7(12):14871–84. doi: 10.18632/oncotarget.7652 (PMC4924758; doi:10.18632/oncotarget.7652)
Supplement: Supplementary file 1 [file oncotarget-07-14871-s001.pdf]

Tumor necrosis factor-α and interferon-γ stimulate MUC16 (CA125) expression in breast, endometrial and ovarian cancers through NFκB

Supplementary Materials

Supplementary Table S1: MUC16, TNFα and IFNγ are present in malignant neoplasms*

Tumor Type	MUC16/CA125 Staining intensity	TNFα Staining intensity	IFNγ Staining intensity
Ovary pt. 1	3	3b	3b
Ovary pt. 2	3	3b	2b
Ovary pt. 3	3	3b	2b
Ovary pt. 4	1	1b	1b
Ovary pt. 5	1	1b	1b
Ovary pt. 6	1	1b	2b
Ovary pt. 7	3	3c	3c
Ovary pt. 8	3	3b	2b
Ovary pt. 9	3	2b	2b
Ovary pt. 10	2	3b	2b
Ovary pt. 11	3	2b	3b
Ovary pt. 12	3	2b	3b
Ovary pt.13	3	3b	2b
Ovary pt. 14	3	3b	3b
Endometrium pt. 1	1	2s	2s
Endometrium pt. 2	0	3b	2s
Endometrium pt. 3	3	3b	2c
Endometrium pt. 4	1	1b	3b
Endometrium pt. 5	3	3c	3b
Endometrium pt. 6	1	3b	3b
Endometrium pt. 7	2	3b	3c
Endometrium pt. 8	3	3b	2b
Endometrium pt. 9	3	3b	3b
Breast pt. 1	3	2b	3c
Breast pt. 2	3	3c	3c
Breast pt. 3	3	3b	3b
Breast pt. 4	3	3b	3b
Breast pt. 5	1	1b	2b
Breast pt. 6	1	3b	2b
Breast pt. 7	3	3b	3
Breast pt. 8	1	3b	3
Breast pt. 9	2	3b	3c
Breast pt. 10	1	1b	2b
Breast pt. 11	1	1b	2c

Breast pt. 12	2	2b	3b
Breast pt. 13	2	2b	3c
Breast pt. 14	3	2b	3b
Breast pt. 15	1	2b	2b
Breast pt. 16	1	2b	2b
Breast pt. 17	1	2b	2b
Breast pt. 18	2	3b	3c
Breast pt. 19	2	3b	2b

*The table shows the results of a tissue array staining for MUC16, TNFα and IFNγ. Each entry represents a sample from different patient. In general, MUC16 is present in areas where TNFα and/or IFNγ are also present. The letters represent staining observed in the following portions of the sections: c = cancer, s = stroma, b = both. MUC16 staining was only observed in the cancers. The numbers 1 to 3 refer to the staining intensity. 1 = low staining level and 3 = high staining level. Examples of staining intensity are shown in Figure 6.

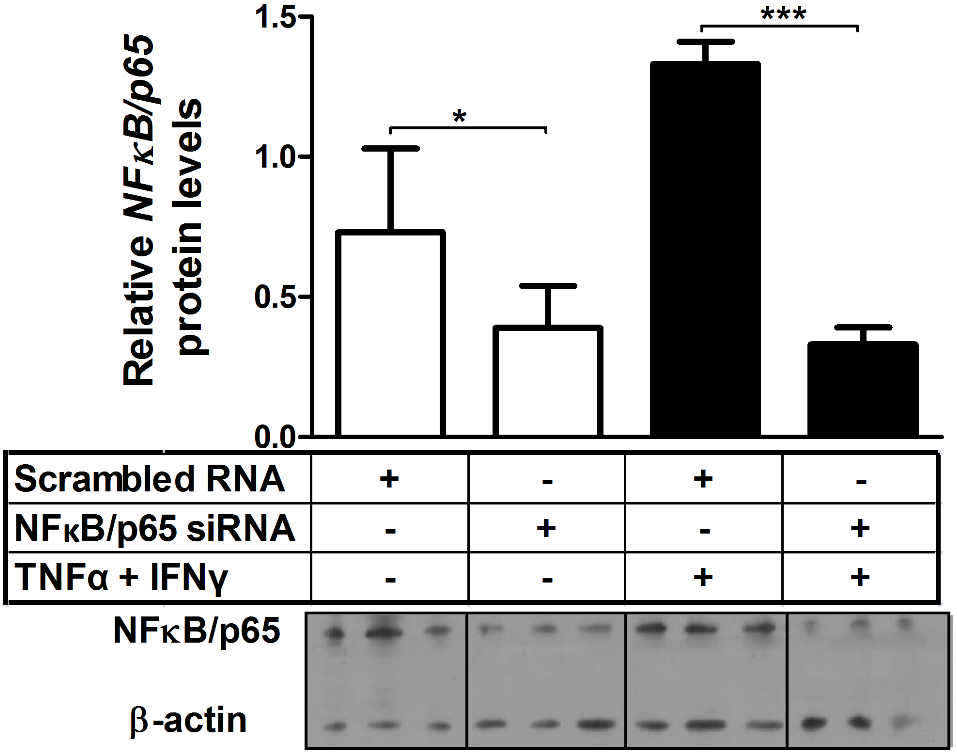

Supplementary Figure S1: Elevation of MUC16 in response to cytokines is attenuated when NFκB/p65 is knocked down by siRNA. MCF-7 cells were treated for 24 h with either scrambled or NFκB siRNA at a final concentration of 50 nM. Cells then were treated for 24 h with either a vehicle control (0.1% [w/v] BSA in PBS), or a combination of TNFα (2.5 ng/ml) plus IFNγ (20 IU). Cell-associated protein then was extracted and the levels of NFκB/*p65* relative to that of β-actin were determined by western blot. The bars and error bars indicate the mean ± SD of triplicate independent determinations in each case. *$p < 0.05$, scrambled vehicle vs. NFκB/p65 siRNA-TNFα + IFNγ and ***$p < 0.001$scrambled TNFα + IFNγ vs. NFκB/p65 siRNA-TNFα + IFNγ.

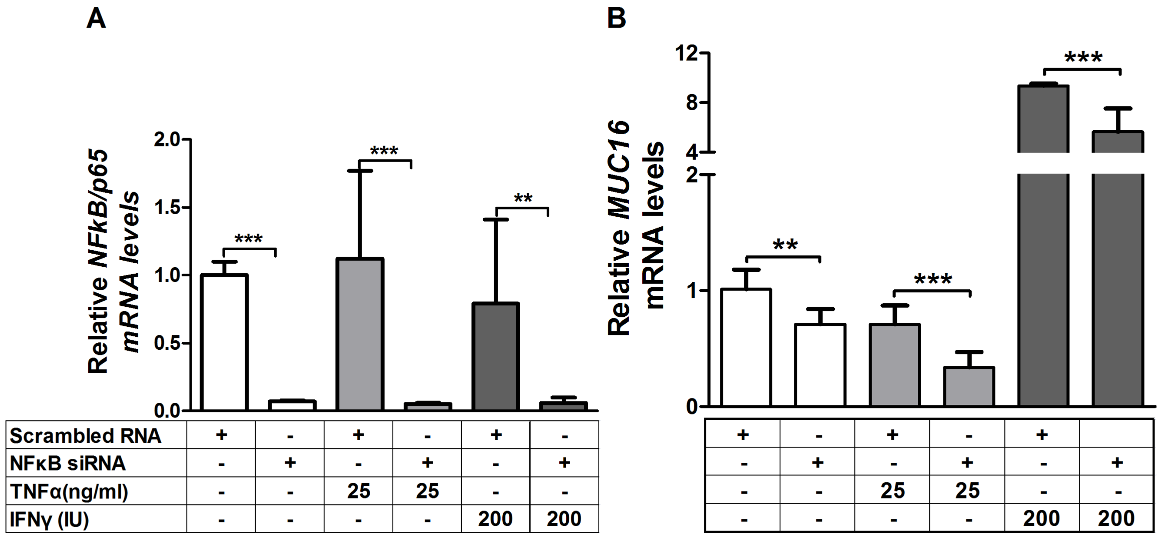

Supplementary Figure S2: ***MUC16*** **mRNA response to high dose of TNFα or IFNγ is attenuated when NFkB/p65 is knocked down by siRNA.** SKOv3-ip cells were treated for 24 h with either scrambled or MUC16 siRNA at a final concentration of 50 nM. Then, cells were treated for 24 h with high doses TNFα (25 ng/ml) or IFNγ (200 IU). The levels of *NFκ*B/*p65* and *MUC16* mRNA relative to that of *ACTB* were determined by qRT-PCR. Values obtained for scrambled vehicle control in each case were set to 1 for comparison. ***$p < 0.001$, scrambled TNFα or IFNγ vs. NFkB/p65 siRNA-TNFα or NFkB/p65 siRNA-IFNγ and ***$p < 0.01$, scrambled vehicle vs. scrambled NFkB/p65 siRNA.

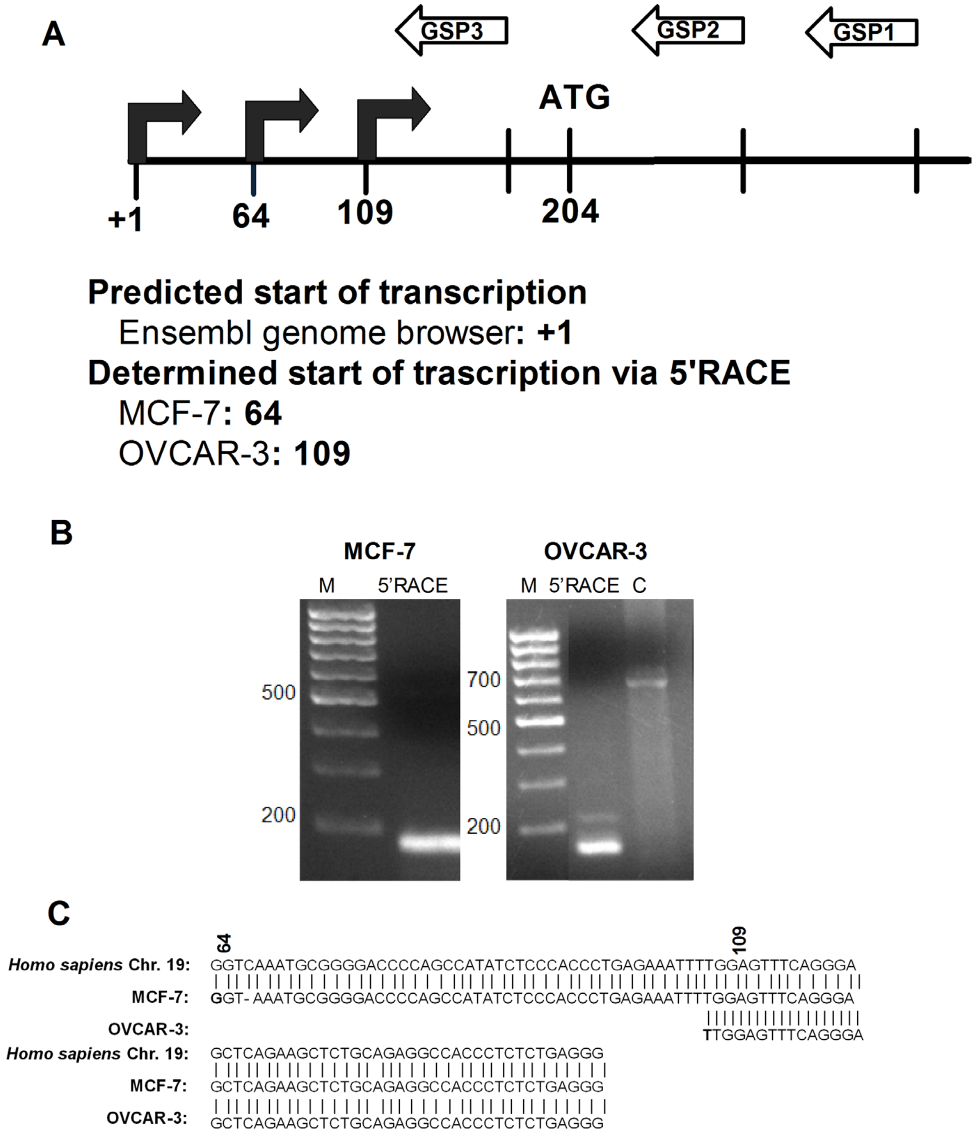

Supplementary Figure S3: Alternative transcriptional start sites of the MUC16 gene. (**A**) The diagram shows two alternative sites of transcription found using 5′RACE in two different cell lines MCF-7 and OVCAR-3. Sites are compared with the start of transcription predicted by the Ensembl genome browser. Diagram also shows the sites of the primers used in 5′ RACE: GSP1, GSP2, GSP3. (**B**) Image shows the bands amplified by PCR used in the 5′ RACE. (**C**) Sequence alignment of human chromosome 19 and the two cell lines MCF-7 and OVCAR-3 in which it is shown how the two cell lines start transcription at sites other than those predicted. Note that MCF-7 shows a nucleotide deletion.

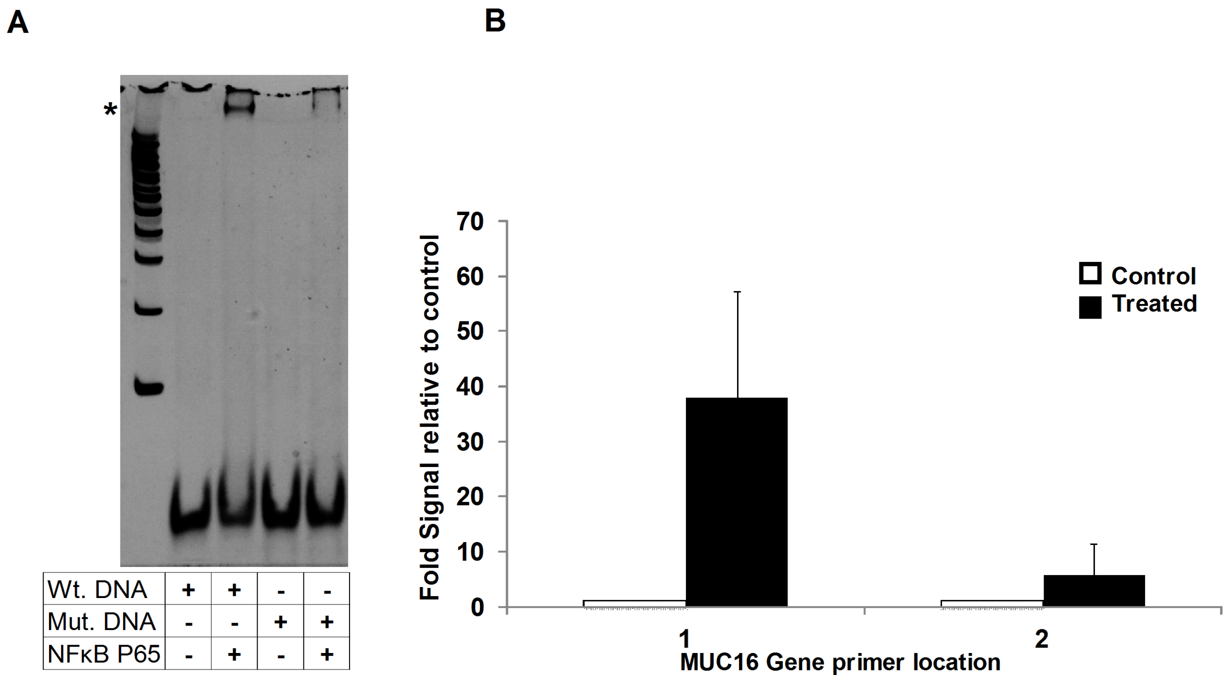

Supplementary Figure S4: Binding and recruitment of NFκB/p65 to the MUC16 promoter. (**A**) An electrophoretic mobility shift assay (EMSA) was performed using unlabeled wild type or mutated NFκB oligos (.15 µM) alone and in combination with the recombinant NFκB/p65 protein (1 µM). Samples were incubating in 5X binding buffer for 40 min and run in a non-denaturing 12% gel followed by gel staining. Gels were imaged using the Carestream Imaging system following the manufacturer's instructions. (**B**) ChIP assay of NFκB/p65 binding to MUC16 promoter was done using MCF7 cells treated in stripped media containing TNFα and IFNγ to a final concentration of 2.5 ng/ml and 20 IU, respectively. Cells were cross-linked and chromatin harvested 4 h following treatment as described in Materials and Methods. Two biological replicates for chromatin extractions were performed and all ChIP assays were performed in triplicate. One µg of chromatin stock was used with 2 µg NFκB antibody as per the standard Chromatrap® ChIP protocol. Two sets of primers (labelled 1 and 2) flanking the MUC16 NFκB site were used as detailed in Materials and Methods. Fold change is above the mean of the background.
